# COVID-19 pandemic effects on orthopaedic surgeons in British Columbia

**DOI:** 10.1186/s13018-021-02283-y

**Published:** 2021-02-26

**Authors:** Maciej J. K. Simon, William D. Regan

**Affiliations:** 1 Department of Orthopaedics, Chan Gunn Pavilion, Allen McGavin Sports Medicine Clinic, University of British Columbia, 2553 Wesbrook Mall, Vancouver, BC V6T1Z3 Canada; 2 Department of Orthopaedics, University Medical Center Hamburg-Eppendorf, Martinistrasse 52, 20246 Hamburg, Germany

**Keywords:** COVID-19 pandemic, Orthopaedic surgeons, British Columbia

## Abstract

**Background:**

COVID-19 was declared a global emergency in the first quarter of 2020. It is has resulted in and continues in over a million deaths worldwide and halted medical systems and particularly elective surgeries worldwide. The aim of this study was to identify the effect of the initial COVID-19 pandemic months on orthopaedic surgeons in British Columbia.

**Methods:**

The study surveyed (June until August 2020) 187 orthopaedic surgeons in British Columbia affiliated with the University of British Columbia or the British Columbia Orthopaedic Association using an online survey to help identify the impact of COVID-19 on patient care, work and personal life.

**Results:**

Eighty-seven out of 187 (46.5%) orthopaedic surgeons participated in the online survey. All types of surgeries were completely cancelled for 23 respondents. Elective surgeries were cancelled for most respondents (in-hospital *n* = 38 and/or ambulatory *n* = 32). Trauma cases were reduced according to 35 respondents. Outpatient clinics were stopped initially and transferred in virtual clinics (telemedicine). Approximately 40% of respondents were afraid of infecting others (patients, family) and 25% admitted to drink more heavily. Ninety percent of respondents reported an income loss of > 15% (range 0–100%).

**Conclusion:**

Orthopaedic surgeons and their patients have been significantly affected by the COVID-19 pandemic. Cancellation of surgeries has created an increased backlog of 32,400 orthopaedic surgeries in British Columbia. However, the COVID-19 pandemic has expedited the implementation of telemedicine, which will be a long-lasting benefit in providing healthcare.

## Background

The COVID-19 pandemic caused by the severe acute respiratory syndrome coronavirus 2 (SARS-CoV-2) has not only resulted in over 41.5 million confirmed cases and over 1,000,000 deaths globally but also caused a major economic crisis prior to the second wave [1]. From the moment on, the World Health Organization (WHO) declared COVID-19 to be a global emergency; multiple restrictions have been passed in order to reduce the spread of the disease and limit deaths [2]. With travel restrictions, quarantine and complete lockdowns of countries, the economy has been slowed down and is sliding into a state of recession. This has also included the healthcare systems globally [3].

In North America, the healthcare systems have severely suffered from this pandemic. Canada has a universal and publicly funded healthcare system, which generally provides all citizens with healthcare services, but it is also known for its prolonged waiting times for elective care procedures [4]. With the declaration of state of emergency in Canada, elective procedures, which are the majority of surgical procedures in orthopaedic surgery, were halted in order to increase ventilation and work-force capacity dedicated to SARS-CoV-2-infected patients since the second half of March 2020 [5, 6]. This public health emergency was therefore implemented in the Province of British Columbia. This has created a significant provincial back-log of 32,400 orthopaedic surgical cases [7–9]. Furthermore, it caused inevitable changes in clinical practice routines and financial circumstances (approximately US$200 million) to succumb the backlog [10].

The University of British Columbia is the only Canadian University with an Orthopaedic Department. In all other provinces, Orthopaedics represents a division of Surgery. The Department offers an expanded provincial wide membership comprising of seven divisions. The aim of this study is to identify how socio-economically, personally and workwise orthopaedic surgeons within the Department of Orthopaedics in British Columbia have been affected by the COVID-19 pandemic and what the perception for the future beholds.

## Methods

A web-based survey was developed and administered using Qualtrics (Qualtrics, Provo, UT, USA), an easy-to-use online survey tool and analysis platform. The survey data is kept secure and is stored and backed up in Canada in order to comply with the British Columbia Freedom of Information and Protection of Privacy Act (FIPPA).

The survey consisted of questions related to the COVID-19 pandemic and its effects on orthopaedic surgery and surgeons in British Columbia, Canada.

The survey was sent to the Orthopaedic Department of the University of British Columbia (UBC) and the British Columbia Orthopaedic Association (BCOA). The survey was distributed July 2020 and closed end of August 2020.

One hundred eighty-seven orthopaedic surgeons were sent an e-mail with the invitation link to take the survey by each secretary. Eighty-seven orthopaedic surgeons of all subspecialties completed the survey and were included in the analysis.

The responses were anonymous and tabulated and reported in aggregate form. Categorial variables were reported as numbers and percentiles.

The survey questions and responses are demonstrated in Tables 1, 2 and 3.

**Table 1:** Participants information and personal effects

	Percentage	Count
**In what region do you currently work?**		
BC—Vancouver Island Coast	17.24%	15
BC—Mainland/Southwest	60.92%	53
BC—Thomsen-Okanagan	11.49%	10
BC—Kootenay	2.30%	2
BC—Cariboo	0.00%	0
BC—North Coast	0.00%	0
BC—Nechako	6.90%	6
BC—Northeast	1.15%	1
Total	100%	87
**How old are you?**		
< 30 years	0.00%	0
30–40 years	18.39%	16
> 40–50 years	37.93%	33
> 50–60 years	19.54%	17
> 60–70 years	21.84%	19
> 70 years	2.30%	2
Total	100%	87
**How would you describe your gender?**		
Male	86.21%	75
Female	9.20%	8
Transgender	1.15%	1
Other	0.00%	0
Prefer not to say	3.45%	3
Total	100%	87
**What division are you in? (select all that apply)**		
Arthroscopy, Joint Preservation and Reconstructive Surgery	21.54%	28
Combined Neurosurgical and Orthopaedic spine	2.31%	3
Comprehensive orthopaedics	23.85%	31
Distal Extremities	11.54%	15
Lower Limb Reconstruction and Oncology	13.08%	17
Orthopaedic Trauma	16.92%	22
Paediatrics Orthopaedics	5.38%	7
Other (please specify)	5.38%	7
Total	100%	130
**How many years have you been practising as a fully-qualified specialist?**		
1–3 years	6.90%	6
3–6 years	10.34%	9
6–10 years	18.39%	16
> 10 years	13.79%	12
> 15 years	16.09%	14
> 20 years	18.39%	16
> 30 years	12.64%	11
> 40 years	3.45%	3
Total	100%	87
**Have you experienced loss of income?**		
No, I am on a salary/alternate payment	9.21%	7
No, I am not on a salary/alternate payment	1.32%	1
Yes, 25% deficit	32.89%	25
Yes, 50% deficit	30.26%	23
Yes, 75% deficit	15.79%	12
Yes, 100% deficit	1.32%	1
Other (please specify)	9.21%	7
Total	100%	76
**Have you sought medical help?**		
Yes	1.37%	1
No	98.63%	72
Total	100%	73
**Have you been drinking more heavily?**		
Yes	25.00%	18
No	75.00%	54
Total	100%	72
**Did you have to self-isolate/quarantine at any point during the COVID-19 pandemic?**		
Yes	20.93%	18
No	79.07%	68
Total	100%	86
**Were there any positive COVID-19 tests (confirmed infection)? (select all that apply)**		
Patient in my hospital	40.82%	60
Patient in my department/division	11.56%	17
Medical staff in my hospital	18.37%	27
Medical staff in my department/division	4.08%	6
Other staff in my hospital	17.01%	25
Other staff in my department/division	1.36%	2
None of the above	6.80%	10
Total	100%	147
**Are you afraid of infecting others? (select all that apply)**		
Yes, family and friends	27.86%	39
Yes, family, but not friends as I do social-distancing	25.71%	36
Yes, patients	25.00%	35
No, I am always wearing personal protective equipment (PPE) at work	10.71%	15
No, I am not a risk group	7.86%	11
Other (please specify)	2.86%	4
Total	100%	140
**If yes to afraid of infecting others, what are your methods of prevention? (select all that apply)**		
I am not staying at home (staying at the hospital, hotel, secondary home, etc.)	1.20%	2
I wash and disinfect my hands more than usual.	31.33%	52
I change my clothes at work more than usual	20.48%	34
I try to self-distance out home from my family/partner	3.01%	5
I wear personal protective equipment (PPE) at home to not infect others	0.00%	0
I avoid physical contact with family/household members	0.60%	1
I am more careful at work than usual	31.93%	53
I have taken time of work, vacation, leave of absence	9.64%	16
Other (please specify)	1.81%	3
Total	100%	166

**Table 2:** Work effects, training and support

	Percentage	Count
**Have you received adequate support for return to work (e.g. proper PPE, protocols)? (select all that apply)**		
Sufficient from the hospital	50.53%	48
Sufficient from the College of Physicians and Surgeons (CPS)	7.37%	7
Received some from the CPS, but not enough	10.53%	10
No, I had to make/buy it on my own	24.21%	23
I work without protocols/personal protective equipment (PPE)	0.00%	0
Other (please specify)	7.37%	7
Total	100%	95
**What measures are put in place in your current work place in order to prevent the spread of COVID-19? (select all that apply)**		
The medical staff has been divided into teams/groups	5.62%	10
Reduced attendance of staff in meetings	17.42%	31
Changing attendance of staff	8.99%	16
Individual protective equipment (e.g. facemasks, gowns, gloves, etc.)	38.20%	68
Home office (scientific research, online education, tele medicine, etc.)	26.40%	47
No prevention measures	1.12%	2
Other (please specify)	2.25%	4
Total	100%	178
**Was there any special COVID-19 training advised for the surgical staff?**		
Yes	56.76%	42
No	43.24%	32
Total	100%	74
**What effects has COVID-19 on your surgeries? (select all that apply)**		
No change	1.04%	2
All surgeries have been cancelled	11.98%	23
Elective hospital in-stay operations have been cancelled	19.79%	38
Elective ambulatory surgeries have been cancelled	16.67%	32
Only selected elective in-stay surgeries can be performed	8.33%	16
Only selected elective ambulatory surgeries can be performed	6.25%	12
Similar amount of trauma cases	7.81%	15
Reduced cases of trauma	18.23%	35
Other (please specify)	9.90%	19
Total	100%	192
**What effects does the COVID-19 pandemic have on your outpatient clinic? (select all that apply)**		
All patients are tested for SARS-CoV-2 before orthopaedic consultation	0.00%	0
All patients have to fill out a screening questionnaire	29.87%	46
All patients are being screened (e.g. temperature check) before an orthopaedic consult	24.68%	38
Only patients with positive symptoms/positive screening (including positive questionnaire) are being tested for SARS-CoV-2	16.88%	26
There are no changes	0.00%	0
Only patients with acute orthopaedic/trauma symptoms (fractures, infections, bone tumours) are allowed to personally come to outpatient clinic	18.83%	29
Other (please specify)	9.74%	15
Total	100%	154
**What effects does the COVID-19 pandemic have on your work as an orthopaedic surgeon? (select all that apply)**		
No effect	0.73%	2
My surgeries have been reduced	22.63%	62
Patient influx has been significantly reduced	21.17%	58
The reason for postponing surgery due to the pandemic is the main topic discussed with patients	8.39%	23
Education and teaching (students, residents, fellows,…) is currently due to the pandemic not possible	9.85%	27
Education and teaching (students, residents, fellows,...) have been switch to online platforms (e.g. Skype, Zoom)	10.58%	29
Increased non-orthopaedic-related work	9.85%	27
Increased administrative work (home office or in hospital)	13.50%	37
Orthopaedic surgeons are put to help in non-orthopaedic related work (e.g. physician-related work)	0.73%	2
Other (please specify)	2.55%	7
Total	100%	274
**Are there any differences in regards to meetings due to the COVID-19 pandemic?**		
No differences in my department/division meetings	0.00%	0
Reduced number of participants in meetings	4.05%	3
There are currently no more meetings	2.70%	2
Everybody is participating in meetings, but with appropriate social distance rules	1.35%	1
Everybody is participating in meetings, but only with wearing proper PPE (personal protective equipment) (e.g. facemasks)	0.00%	0
Everybody is participating in meetings, but with appropriate social distance rules and wearing proper PPE (personal protective equipment) (e.g. facemasks)	1.35%	1
Meetings are exclusively switched to online video-conference platforms (e.g. Skype, Zoom)	86.49%	64
Other (please specify)	4.05%	3
Total	100%	74
**Are you offering consulting services with telemedicine? (select all that apply)**		
Web-based telemedicine/virtual clinics (FaceTime, Google Chat, Skype, Zoom, etc.)	44.88%	57
Telephone	51.18%	65
No services offered	2.36%	3
Other (please specify)	1.57%	2
Total	100%	127

**Table 3:** Future perspectives and effects of COVID-19

	Percentage	Count
**How long do you think the COVID-19 pandemic will reduce your “normal” clinical/surgical routine?**		
2–4 weeks	0.00%	0
5–8 weeks	1.35%	1
9–12 weeks	4.05%	3
3–6 months	20.27%	15
6–9 months	5.41%	4
9–12 months	8.11%	6
> 12 months	45.95%	34
Indefinitely	14.86%	11
Total	100%	74
**Do you think there will be a vaccine before 2021 or adequate treatment?**		
Yes	16.22%	12
No	83.78%	62
Total	100%	74
**Do you think we will adapt herd immunity?**		
Yes	22.97%	17
No	77.03%	57
Total	100%	74
**Do you think if there is herd immunity should surgeons 60 years of age and older limit their surgical scope?**		
Yes	27.03%	20
No	72.97%	54
Total	100%	74
**What effects has the COVID-19 pandemic on you personally?**		
I work and do surgeries as usual	22.58%	21
I am currently not working due to postponement of elective surgeries, but do scientific research, administrative tasks, virtual clinics, etc.	4.30%	4
I am currently not doing surgeries due to personal exposition to COVID-19, or travel quarantine	0.00%	0
I am currently doing trauma cases only	4.30%	4
I am currently not doing surgeries because I am 60 years of age or older	4.30%	4
My productivity will be compromised moving forward	51.61%	48
Other (please specify)	12.90%	12
Total	100%	93
**Do you feel more or less optimistic about the future?**		
Yes	58.90%	43
No	41.10%	30
Total	100%	73
**If you feel more or less optimistic about the future, do think this will be a permeant change?**		
Yes	65.12%	28
No	34.88%	15
Total	100%	43
**Will you have to work longer before you retire as a result of COVID-19?**		
Yes	36.99%	27
No	63.01%	46
Total	100%	73
**If you have to work longer before you retire due to COVID-19, how much longer?**		
1–2 years	44.44%	12
2–5 years	33.33%	9
> 5 years	22.22%	6
Total	100%	27
**Will you be including telemedicine services in your future practices from now on?**		
Yes	91.78%	67
No	8.22%	6
Total	100%	73
**If you will be including telemedicine services, when do you see this applicable?**		
All situations	44.62%	29
Only initial long-distance consults	20.00%	13
First postoperative follow-up	12.31%	8
Other (please specify)	23.08%	15
Total	100%	65

## Results

Eighty-seven of 187 orthopaedic surgeons (46.5%) participated to the survey. Majority of questions were single response and others allowed multiple answers. The survey results are demonstrated in Tables 1, 2 and 3. Data of participant information and personal effects is demonstrated in Table 1. Most respondents (60.9%) are employed in the mainland/southwest (metropolitan region of Vancouver) of British Columbia. The majority are male (86.2%) and aged between 40 and 50 years of age (37.9%).

Geographical representation was negatively skewed with regard to orthopaedic surgeons practising in the more isolated communities of The Kootneys, Cariboo and North Coast regions who collectively provided limited response to the questionnaire. This was in spite of repeated attempts to engage these surgeons. Their non-compliance issues have not been identified in spite of efforts to do so. The province-wide responders were distributed within the following sub-specialties of comprehensive orthopaedics (23.9%), arthroscopic and joint preservation surgery (21.5%) and orthopaedic trauma (16.9%). Currently, about 90% of respondents have experienced an income deficit ranging between 15 and 100%. The others (*n* = 7) were on a salary payment. One participant stated to have sought medical help to deal with mood disorder secondary to income loss. Alcohol consumption has increased in 25% of survey participants.

Eighteen responders had to isolate/quarantine during the COVID-19 pandemic as they had been exposed to a positive COVID-19 case or co-worker or had positive test results themselves (Table 1). The majority (78.6%) were afraid of infecting their family, patients and friends. Many respondents say that they are more careful now at work, particularly washing and disinfecting hands (*n* = 52), and they change their work clothes mare than usual (*n* = 34).

Table 2 demonstrates participants how their work was affected, what training and support they received. A modest majority (57.9%) stated that they received adequate support to return to work from hospitals or the Royal College (Table 2). This also implies that support was inadequate in 42.1% which is troubling. Special COVID-19 advisory trainings for the surgical staff were implemented according to 56.8% of respondents. Further measures to limit the spread of COVID-19 included increase in personal protection (e.g. facemasks, gowns, gloves; *n* = 68), increased computer-based work (*n* = 47) and reduced attendance in necessary meetings (*n* = 31). Meetings were switched to and performed on online platforms (86.5%).

The majority of respondents stated that, during the study period (March–August 2020), their practice has been affected with full elective ambulatory (*n* = 32) and in-patient surgeries (*n* = 38) had been cancelled and also after hours trauma surgeries were reduced (Table 2). Two respondents did not experience an effect on their surgeries. Many outpatient clinics have now implemented COVID-19 screening questionnaire (*n* = 46) and temperature checks (*n* = 38). Only mandatory visits were scheduled for in-person consults. Generally, the respondents said that patient influx (*n* = 58) and orthopaedic surgeries (*n* = 62) were reduced (Table 2). Thirty-seven respondents stated that their administrative work increased with the COVID-19 pandemic. Two orthopaedic surgeons experienced no effect on their work.

Table 3 shows the respondents results on the effects of COVID-19 and their future perspectives. Most of the respondents (60.8%) believe that the COVID-19 pandemic will affect the routine work (clinics and surgeries) more than 12 months or indefinitely (Table 3). Less than 20% believe that there will be a vaccine before 2021 and 22.9% of respondents believe that the community will adapt to herd immunity.

Most respondents (51.6%) believe that COVID-19 will compromise their productivity in the future. Furthermore, many respondents (58.9%) are less optimistic about the future as they believe there will be a permanent change (65.1%). This reflects in 37% of respondents believing that they will have to work longer before retiring. Over 91% of respondents stated they will implement telemedicine into their future practice pattern for various scenarios.

Cross-sectional analysis of age, region and division compared against loss of income demonstrated that the majority of respondents (*n* = 60; 69%) had a loss of income between 25 and 75% throughout all regions and age groups. The division of arthroscopy was affected the most (38.3%), followed by comprehensive orthopaedics with 30% and 11.7% each for distal extremities and lower limb reconstruction throughout all regions. Orthopaedic paediatrics and the division of combined neurosurgery and orthopaedic spine surgery had with 5 respondents the least loss amongst all divisions.

Despite the loss of income, 54% of respondents feel optimistic about the future. The other group of respondents, who feels less optimistic, has been drinking more heavily in 21% cases. Independent of future optimism, 22% of all respondents with an income loss were drinking more during the COVID-19 pandemic. Overall, one respondent sought medical help having income loss of 50% and being less optimistic but was not drinking more heavily.

## Discussion

The COVID-19 pandemic has tremendously influenced the work of orthopaedic surgeons in British Columbia. All orthopaedic subspecialties have experienced work-related and personal compromise uniformly distributed within the province by the pandemic. Elective surgeries (ambulatory and in-stay) have either been significantly reduced or completely cancelled as a result of the pandemic. This has led to a significant financial loss in about 90% of respondents. All other respondents were on a salary payment. The northern interior, Kootney, and isolated North coast community surgeons were poorly represented in the questionnaire secondary to poor compliance. This had nothing to do with government policy as all imposed health care constraints were province wide. Hence, these surgeons would not be busier nor more burdened with additional teaching nor administration. They are all fee for service as well; hence, their financial picture would reflect others in that category. Further analysis will be important in the future. It may be simply that these surgeons enjoy the isolation of their work and lifestyle which encompasses all aspects of their lives.

With the COVID-19 being declared a global pandemic and the halt of elective surgery in Canada in mid-March, orthopaedic surgeons were forced to stop planned non-urgent surgeries in order to reduce the spread of COVID-19 [2, 5, 6], and prevent shortages of ventilators, personal protective equipment (PPE) and blood products as particularly observed in US-based hospital [11–13]. Only, urgent surgeries, such as life- or limb-threatening trauma and cancer- or infection-related cases, which could not be postponed, were performed by most surgeons during the first 3 months after stoppage of elective surgery. That explains why elective surgeries such as arthroscopic procedures were cancelled in British Columbia, and since this subdivision usually works on a fee for service basis, these orthopaedic surgeons were most heavily affected with loss of income. And on the other hand, the division of combined neurosurgery and orthopaedic spine was the least income-affected subdivision, as three out of five have a fixed salary or alternate payment. In addition, their patient population, by nature of their injury pattern, would require more urgent and emergent care.

During the initial pandemic phase, orthopaedic outpatient clinics were reduced or cancelled. The patient flow changed during the initial COVID-19 phase. Less trauma cases were registered at emergency departments in Italy, which lead to a percentage increase of fragility fractures, and an overall change of patient flow in emergency departments [14]. This cause and reallocation of resources to COVID-19 patients lead to a tremendous decrease in outpatient clinics, admissions and surgeries [15]. Therefore, new measures such as telemedicine or telephone consults were implemented and rapidly have become the “new norm”. Telemedicine is currently employed by most orthopaedic surgeons in British Columbia and will remain part of their practice in the future (92.9%). The use of telemedicine is extremely useful as a screening tool but it still will not replace a proper clinical examination which is needed in many orthopaedic disorders. However, telemedicine will likely remain an excellent screening tool to manage the vast area of British Columbia with approximately 1,000,000 km^2^ where approximately half of its population live in metropolitan Vancouver and the other half dispersed throughout the province [16]. Before COVID-19, telemedicine was scarcely more used in Europe, Korea or Japan, but it has been more prevalent in the USA [17, 18]. However, since COVID-19, it has made a significant jump in its use and place in healthcare [19]. Therefore, telemedicine will most likely have a greater impact in the delivery of healthcare following the COVID-19 pandemic, particularly as a premiere screening tool as seen in British Columbia and worldwide [20].

Canada’s universal healthcare system is widely regarded as a pillar of Canadian society, but the limited operating room (OR) capacity and hence the extensive wait times for elective surgery already prior COVID-19 remain controversial [4]. With the cessation of elective surgery for approximately 3 months (mid-March to mid-June), a significant large backlog of more than 400,000 operations has been created throughout Canada [21]. A modelling study estimated for those 3 months that there is an overall backlog of 148,364 surgeries (all specialties) in the Province of Ontario leading to an average weekly increase of 11,413 surgeries [22]. Furthermore, the backlog clearance for this particular time period has been estimated to about 84 weeks. This model can be applied to other provinces and territories in Canada, when adapted accordingly to its population, OR times, ward and ICU beds and healthcare providers. In British Columbia, the backlog is estimated to have increased by 32,400 cases which will cost US$200 million extra per year and take about 2 years to work through [9, 10]. This model is particularly to Canada; however, operating times and postponements of surgeries have been reduced in many countries around the globe during the initial phase of the pandemic [23].

In addition to significant impact on clinical patient care, undergraduate or residency training was inflicted [24]. New teaching and training modalities had to be implemented and many new virtual tools were incorporated more rapidly due to the abnormal circumstances of the COVID-19 pandemic [25, 26]. Furthermore, health-care workers involved in the immediate COVID-19 patient care and others reallocated to new duties, but also the staff remaining in their previous roles, can face increased psychological strain at work as described by Wong et al. [27]. This has to be recognized by hospitals and employers in order to maintain mental well-being of the healthcare staff.

The survey was completed as British Columbia, Canada a Northern hemisphere country which was in the summer period and COVID-19 spread and infections numbers were lower than during the initial period post global-pandemic status. However, the serious negative implications this short time period has had on the orthopaedic surgeons personally and financially, and particularly medically for their patients and treatment postponements will test the resolve of surgeons and patients alike when the fall and winter months come upon us with a second wave.

## Conclusions

The survey amongst orthopaedic surgeons in British Columbia demonstrates that the early months of the global COVID-19 pandemic have had a significant effect on elective surgeries and work routines. This affected surgeries and patients physically and emotionally. Orthopaedic surgeons have had to alter their practises whilst most have lost significant income (15–75%). The impact has affected those most with elective practises. Psychological repercussions have been realized. The implementation of telemedicine has been expedited with the pandemic and has secured a place in the future of healthcare treatment amongst orthopaedic surgeons. The surgical backlog of elective surgeries (32,400 in British Columbia) will be difficult to overcome, and the return to clinical/surgical routine will take according to the majority of orthopaedic surgeons more than 12 months.

## Data Availability

All data has been presented in this manuscript. The data is anonymous. Further data is not available.
